# Investigation of genes expression of the JAK/STAT signalling pathway and AMPs in the presence of *Borrelia* spirochetes in *Ixodes ricinus*

**DOI:** 10.1038/s41598-025-87506-6

**Published:** 2025-01-22

**Authors:** Magdalena Szczotko, Sandra Antunes, Ana Domingos, Małgorzata Dmitryjuk

**Affiliations:** 1 Department of Biochemistry, Faculty of Biology and Biotechnology, University of Warmia and Mazury in Olsztyn, Oczapowskiego 1A, Olsztyn, 10-719 Poland; 2 Global Health and Tropical Medicine (GHTM), Associate Laboratory in Translation and Innovation Towards Global Health (LA-REAL), Instituto de Higiene e Medicina Tropical (IHMT), Universidade NOVA de Lisboa (UNL), Rua da Junqueira 100, Lisbon, 1349-008 Portugal

**Keywords:** Gene expression, Parasitology

## Abstract

Multicellular animals need to control the spread of invading pathogens. This is a particular challenge for blood-feeding vectors such as ticks, which ingest large amounts of blood potentially laden with harmful microorganisms. Ticks have a basic innate immune system and protect themselves from infection through innate immune responses involving pathways such as Janus kinase (JAK) or the signalling transducer activator of transcription (STAT). Direct antimicrobial defence occurs through the rapid synthesis of numerous antimicrobial agents including antimicrobial peptides (AMPs). The tick *Ixodes ricinus* is one of the main vectors of the Lyme disease pathogen, the spirochete *Borrelia burgdorferi* sensu lato. Data suggest that the JAK/STAT signalling pathway controls the expression of AMPs and regulates the infection of the pathogen in the tick body. The innate immune system during the off-host period keeps the level of spirochete infection in check. Spirochetes may influence the innate immune response in ticks. Therefore, the aim of this study was to analyse the expression of the genes related to the JAK/STAT pathway and selected AMPs in questing ticks in which *B. burgorferi* s.l. was detected. In the ticks infected with spirochetes, overexpression of genes related to the JAK/STAT signalling pathway was observed in the case of *STAM* and *SOCS* genes. AMPs genes such as *def1*, *ric*, *lzs* were overexpressed with different expression patterns. The results obtained suggest that AMPs may be involved in infection management in ticks.

**Supplementary Information:**

The online version contains supplementary material available at 10.1038/s41598-025-87506-6.

## Introduction

The hard tick, *Ixodes ricinus* (Acari: Ixodidae), which is widely distributed in northern Europe, including Poland^1,2^ is known to transmit a variety of pathogens that are important in both the medical and veterinary fields^3^, such as tick-borne encephalitis virus, *Anaplasma phagocytophilum*, *Rickettsia* spp. and parasitic protozoa of the genus *Babesia* spp. Firstly, ticks are known to transmit bacteria of the *Borrelia burgdorferi* sensu lato (s.l.) complex, which are spirochetes that cause Lyme disease (LD) and are spread by *Ixodes* spp. ticks. It is considered the most common tick-borne infectious disease in North America and in Eurasian countries with temperate climates^4^. More than 85,000 cases of LD are documented each year, with the highest prevalence observed in Central European countries such as the Czech Republic, Estonia, Lithuania, Slovakia, and Poland^2^.

As in other arthropods, the defence of ticks is based on innate, non-specific immunity^5^. The cellular response in ticks is associated with hemocytes, which perform various functions such as phagocytosis, nodule formation and encapsulation^6^. The regulation of the humoral response in ticks occurs via four main signalling pathways: Nuclear factor-kappa B/Toll (NF-κB/Toll), c-Jun N-terminal kinase (JNK) immunodeficiency (IMD) and Janus kinase/signal transducer and activator of transcription (JAK/STAT)^6–8^. Although the JAK/STAT signalling pathway in the tick remains poorly understood, recent research has shown that it is indeed functional, suggesting its important role in pathogen control^7–9^. The JAK/STAT pathway in ticks is activated in response to bacterial or protozoan pathogens. The expression of antimicrobial peptides (AMPs) in the salivary glands and hemocytes of *Ixodes scapularis* is controlled by the JAK/STAT pathway, which can both enhance and inhibit the regulation of infections^6,10,11^. In *I. scapularis*, the JAK/STAT pathway plays a crucial role in mediating signals between the gut microbiota and pathogen colonisation^6,11–13^.

Tick AMPs include lysosomes, cystatins, defensins and other novel AMPs that have been isolated from numerous tick species^14^. AMPs are an immediate response to danger, rapidly inhibit microbial replication and are an essential component of the protective mechanism^15,16^. AMPs are naturally occurring molecules that can kill multidrug-resistant (MDR) bacterial strains. Therefore, clinical interest in these peptides has increased. The prevalence of microbial MDR pathogens in the environment is putting pressure to discover a new class of antibiotics^17,18^.

A significant increase in the expression of defensin-like genes was observed in *I. ricinus* individuals infected with *B. burgdorferi*. Similar results were observed in studies with the ticks *Dermacentor reticulatus* and *Dermacentor variabilis*^19–21^. Furthermore, the data suggest that the JAK/STAT signalling pathway in *I. scapularis* ticks plays a crucial role in the regulation of *A. phagocytophilum* infection in ticks by controlling the expression of AMPs^11^. Previous studies of interactions between ticks and tick-borne pathogens (TBPs) have shown that both the IMD and JAK/STAT signalling pathways play a central role in the control of bacterial infections by organisms such as *B. burgdorferi*, *A. marginale* and *A. phagocytophilum*^7^. For this reason, the interactions between the spirochetes collected during host blood feeding and the innate immune system of ticks remain of interest to researchers. Understanding the mechanisms of infection control and its suppression in ticks forms the basis for the search for effective therapeutics against LD.

**Fig. 1:**
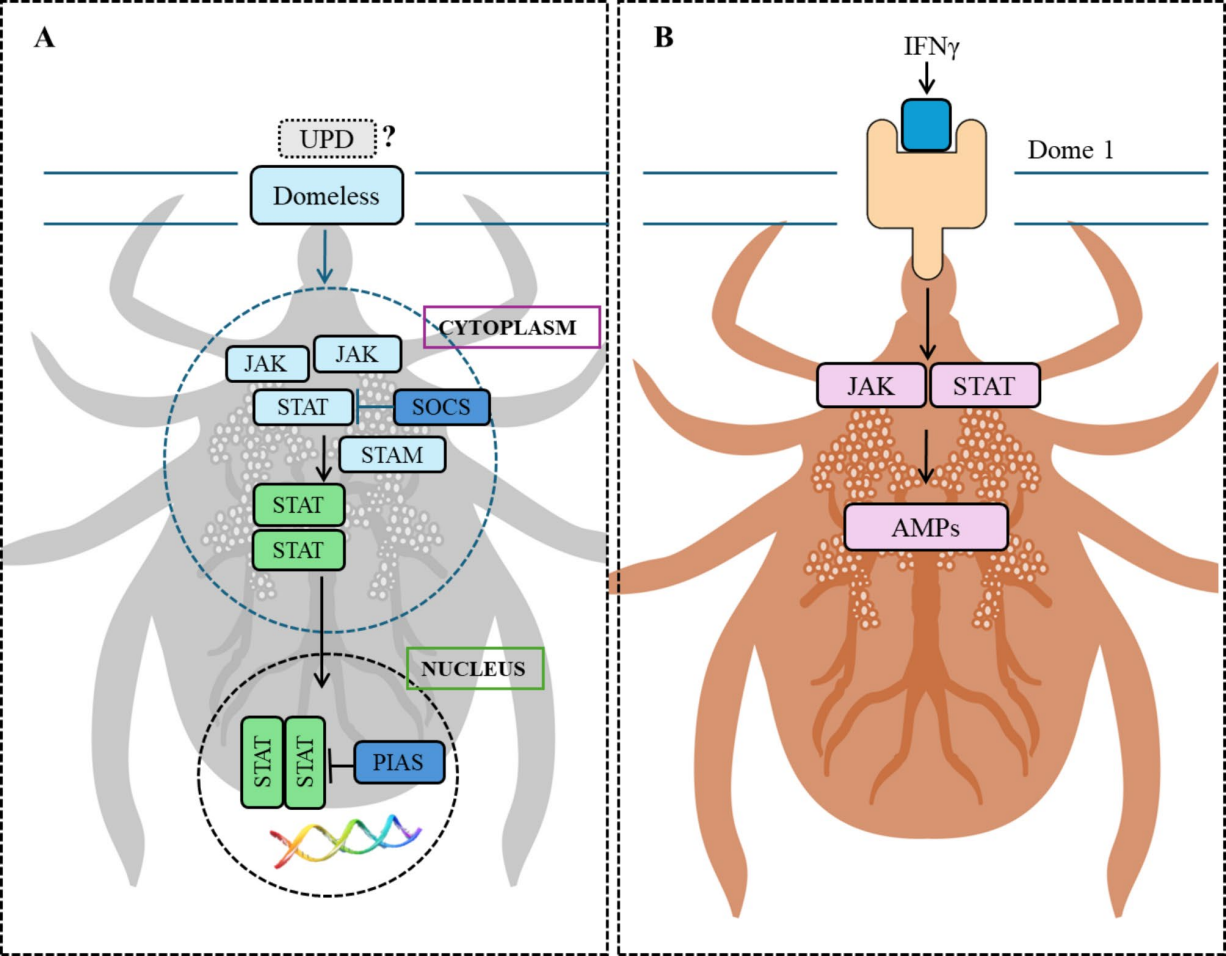
(**A**) The components of the JAK/STAT signalling pathway: the transmembrane cytokine receptor Domeless, the tyrosine kinase (JAK), the transcription factor (STAT), the signal-transducing adaptor molecule (STAM) and the inhibitor proteins: protein inhibitor of activated STAT (PIAS) and suppressor of cytokine signalling (SOCS), UPD gene - has not been identified in ticks according Fogaca et al. 2021^7^ (**B**) Activation of JAK – STAT pathway in feeding ticks according Rana et al. 2023^24^ (Made with SciPainter).

It is widely known that there are three key parts of JAK/STAT signalling: Janus kinases (JAKs), signal transducers and activators of transcription proteins (STATs), and receptors that bind chemical signals^22^. Fogaça et al.^7^ have summarised the conserved components of the JAK/STAT pathway as follows: the transmembrane cytokine receptor Domeless, the tyrosine kinase JAK, the transcription factor STAT, the signal-transducing adaptor molecule (STAM) and the inhibitor proteins PIAS (protein inhibitor of activated STAT) and SOCS (suppressor of cytokine signalling). The ligand of the Domeless receptor (UPD gene) has not yet been identified in ticks (Fig. 1A). Recently, *I. scapularis* ticks have been shown to ingest *Borrelia* and IFN-γ (interferon gamma) during a blood meal from infected hosts^9^. Rana et al.^23^ identified an *I. scapularis* receptor, Dome1, which has high selectivity and affinity for IFN-γ in vertebrates (mouse, human and bird). Unlike Dome orthologues in other arthropods, including non-*Ixodes* ticks, Dome1 has unique extracellular regions like those of cytokine receptors in vertebrates. Dome1 is co-localised with IFN-γ on the luminal surface of the tick endemic epithelium and is induced by ingested IFN-γ, regulating microbicidal responses mediated by the JAK–STAT pathway. Dome1 enhances the immune responses of the tick and is essential for the development of *I. scapularis* (Fig. 1B)^23^.

The role of signalling pathways and innate defence mechanisms in ticks that feed on infected host blood seems clear. On the other hand, taming the infection and establishing the balance between spirochetes and tick tissue seems inspiring in times of silence. Innate immune system during off-host period keeps the level of spirochete infection in check. Spirochetes, possibly have impact on the innate immune response in ticks.

Defensins are conserved defence mechanisms and one of the most important components of the innate immunity of ticks. We decided to test defensin-1 *(def1)*, defensin-2 *(def2)* and ricinusin *(ric)*, a tick AMP belonging to the defensin group^24^. Lyzosyme *(lzs)* appears to be an equally important AMP. It has been shown to have a borreliacidal effect in combination with varisin, a defensin identified in *Dermacentor variabili*s^25^. Our study is therefore based on the hypothesis that the expression of genes related to the JAK/STAT signalling pathway and AMPs (*def1*, *def2*, *ric*, *lzs*) in ticks in the natural environment would increase in response to the presence of LD spirochetes. The aim of the present study was to analyse the gene expression of components of the JAK/STAT immune signalling pathway in questing female *I. ricinus* ticks infected with *B. burgdorferi* s.l. spirochetes and to investigate the selected AMPs expression patterns in different *I. ricinus* tissues. The study was conducted to identify gaps and elucidate the basic immune mechanisms in ticks in the presence of *Borrelia burgdorferi* s.l. spirochetes in *I. ricinus* ticks.

## Materials and methods

### Ticks collection

The study material for the present study consisted of 50 observably non-fed *I. ricinus* adult females. The individuals were collected in urban areas of the Warmia and Mazury region in north-eastern Poland in the period from April to October 2023. The ticks were collected in the housing estate of Olsztyn (Pieczewo) between 9 am and 4 pm using the standard flagging method^26^. The ticks were stored in STAY RNA™ buffer (A&A Biotechnology, Gdynia, Poland) until analysis. All individuals were identified by species, sex and developmental stage using a taxonomic key^27^. Ticks classified as female *I. ricinus* were further analysed.

### RNA extraction

The preserved ticks were rinsed three times in a sterile 0.9% NaCl solution. Dissection was performed under the microscope using sterile forceps and a scalpel in a sterile water drop. During dissection, three tissue groups of the examined individuals were distinguished from the samples: salivary glands (SG), midgut (MG) and the remaining tissues (REST). The genetic material of the 150 tissue samples was extracted individually using the NZYol reagent (NZYTech, Lisbon, Portugal) according to the manufacturer’s instructions. The RNA concentration obtained was checked with NanoDrop (Thermo Fisher Scientific, Waltham, MA, USA).

### cDNA synthesis

cDNA was obtained from RNA extracted from tick tissue using the TranScriba kit (A&A Biotechnology, Gdańsk, Poland). The quality and quantity of cDNA after reverse transcription were determined using NanoDrop™ (Thermo Fisher Scientific, Waltham, Massachusetts, USA). Tecan Infinite M200 (Tecan Group Ltd., Männedorf, Switzerland) was also used to confirm the results obtained. cDNA was synthesised using the TranScriba Kit (A&A Biotechnology, Gdańsk, Poland) with an equal amount of RNA (1 µg). cDNA was diluted with nuclease-free water to achieve a concentration of 100 ng/µl.

### *B. burgdorferi* s.l. detection

The cDNA tick samples of the test groups were subjected to a quantitative real-time polymerase chain reaction (qPCR). All three tissues (MG, SG, REST) of each individual were tested for the presence of *B. burgdorferi* s.l. Specifically, the 23S RNA sequence of *B. burgdorferi* s.l. was analysed using the primer pair Bb23Sf (5`-CGA GTC TTA AAA GGG CGA TTT AGT-3`) and Bb23Sr (5`-GCT TCA GCC TGG CCA TAA ATA G-3`) to obtain a 75-bp fragment with a Bb23Sp TaqMan probe (5`-AGA TGT GGT AGA CCC GAA GCC GGTG-3`) labelled at the 5′ end with FAM and at the 3′ end with TAMRA. The 23S rRNA gene is highly conserved among *Borrelia* species. The method has been shown to be sensitive and specific for the detection of LD spirochaetes and is routinely used in scientific research^28–31^. The reactions were performed in triplicate. The reaction mixture for spirochaete detection consisted of: 1 µl cDNA (100 ng), 5 µl RT PCR mix sample (A&A Biotechnology, Gdańsk, Poland), 0.2 µl probe and 0.5 µl of 10 µmol per primer. The samples were adjusted to a final volume of 10 µl with nuclease-free water. PCR was performed according to the protocol described by Cicculi et al.^28^.

### Genes expression assays

**Fig. 2:**
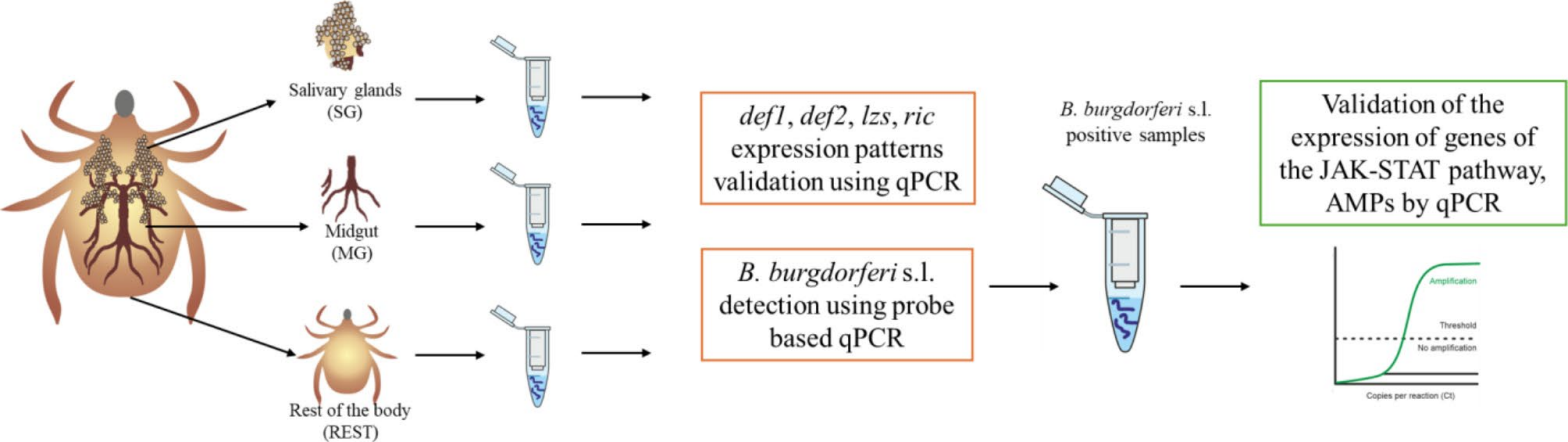
Experimental study scheme (Made with SciPainter).

Five genes involved in the tick JAK/STAT signalling pathway - tyrosine kinase (*JAK)*, transcription factor (*STAT)*, transducing adaptor molecule (*STAM*), suppressor of cytokine signalling (*SOCS)*, protein inhibitor of activated STAT (*PIAS)* and four genes encoding different AMPs – defensin-1 (*def1)*, defensin-2 (*def2*)^17^, lysozyme (*lzs*), ricinusin (*ric*) were selected for this study (Table S1 – Supplementary Data). The primers used were designed and subjected to an optimisation test to determine the optimal annealing temperature and concentration. A standard curve with a sample series dilution of the target template was used to estimate qPCR efficiency. Each standard curve contained 6 concentrations of the target template prepared by 10-fold dilution. The qPCR efficiency varied between 98% and 100% for each assay. The qPCR was performed in triplicate using the QuantStudio™ 3 Real-Time PCR System from Applied Biosystems (Thermo Fisher Scientific, Waltham, MA, USA). The reaction mixture consisted of 1 µl cDNA (100 ng), 5 µl RT PCR Mix SYBR^®^ (A&A Biotechnology, Gdańsk, Poland) and 0.2 µl ROX (5-carboxy-X-rhodamine) reference dye, 0.5 µl of 10 µmol each primer (Table S1 - Supplementary Data) and the volume was made up to 10 µl with nuclease-free water. Results were presented as fold changes in gene expression normalised to two endogenous reference genes, actin and elongation factor (ACT, EF) and calculated using the 2 − ΔΔCT method^17,32,33^ (Fig. 2). The components of the JAK/STAT immune signalling pathway (*JAK*, *STAT*, *STAM*, *SOCS*, *PIAS*) were examined in eight samples with confirmed presence of *B. burgdorferi*^11^. Apart from the reference genes, the results were normalised to the controls of the each tested tissue of the tissue, SG - salivary glands, MG - midgut, REST - the remaining tissue after dissection (relative quantification (RQ) = 1; tissues from eight ticks without confirmed presence of spirochetes).

### Statistical analysis

The data were analysed using GraphPad Prism 8.0 (GraphPad Software, CA, USA) and Statistica 13.3 (StatSoft Inc., Tulsa, Oklahoma, USA). The ΔCq values obtained were subjected to statistical analysis. The normality of the ΔCq values obtained was confirmed using the Shapiro–Wilk test^34^. Analysis of variance (ANOVA) or its non-parametric equivalent was used to assess the difference between the means of the compared groups. P values < 0.05 were considered statistically significant.

## Results

***B. burgdorferi s.l. detection***. *B. burgdorferi* were detected in the genetic material of eight *I. ricinus* individuals. The overall prevalence was 16%. The cDNA of each dissected tissue (24 samples) was subjected to JAK/STAT pathway analysis. Eight ticks showing negative results of *B. burgdorferi* s.l. were randomly selected and used as controls (24 samples from 8 individuals).

**Fig. 3:**
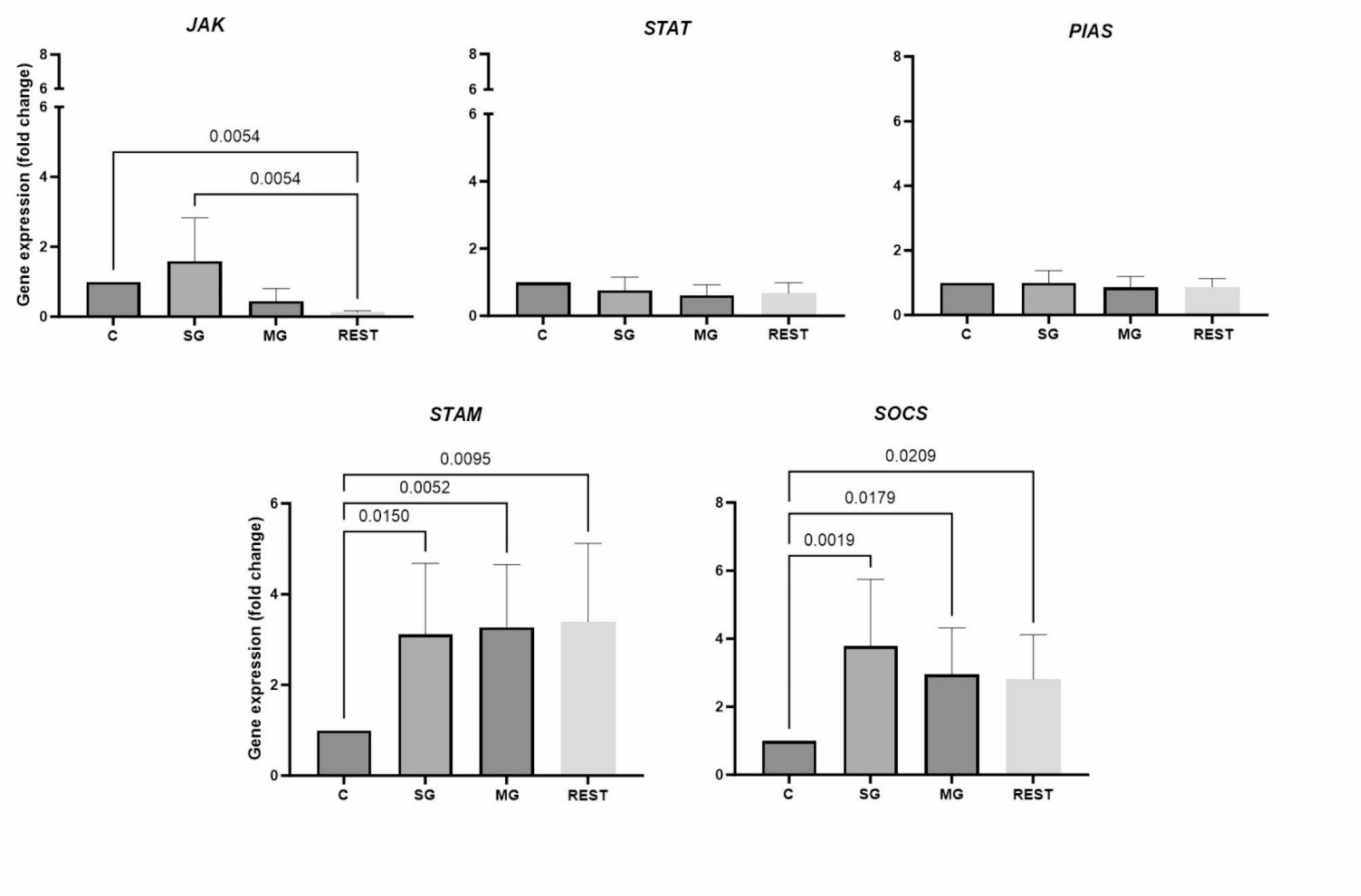
Differences in *JAK*, *STAT*, *PIAS*, *STAM* and *SOCS* expression levels in *I. ricinus* ticks with confirmed presence of *B. burgdorferi* s.l. The results shown are means and standard deviations of gene expression levels. Results in control and ticks were analysed by parametric or non-parametric ANOVA (* *p* < 0.05) and normalised to two endogenous reference genes, actin and elongation factor, and to the relative controls (relative quantification (RQ) = 1; *B. burgdorferi* s.l. negative tissue). C- relative control of the each tissue tested, SG - salivary glands, MG - midgut, REST - the remaining tissue after dissection.

**JAK/STAT pathway genes expression in ticks**. No changes were detected in the gene expression of *STAT*, *PIAS* in individual *B. burgdorferii* s.l. − positive ticks. Significant changes were observed in the *JAK* (down-regulation of its expression in rest of the tissues), *STAM* and *SOCS* genes (Fig. 3).

*JAK* gene expression was downregulated in tissues other than the midgut and salivary glands (8.3-fold change, *p* = 0.0054). Comparison of fold change between the tissues examined showed significant differences between the salivary glands and the rest of the body, with expression being 13.3-fold higher (*p* = 0.0054) in the salivary glands. The *STAM* gene was upregulated in all tissues examined. Gene expression was 3.11-fold higher in the salivary glands (*p* = 0.0150), 3.27-fold higher in the midgut (*p* = 0.0052) and 3.39-fold higher in the rest of the tissues (*p* = 0.0095). The *SOCS* gene was upregulated 3.79-fold (*p* = 0.0019) in the salivary glands 2.96-fold (*p* = 0.079) in the midgut and 2.8-fold (*p* = 0.0209) in the rest of the body (Fig. 3).

**Fig. 4:**
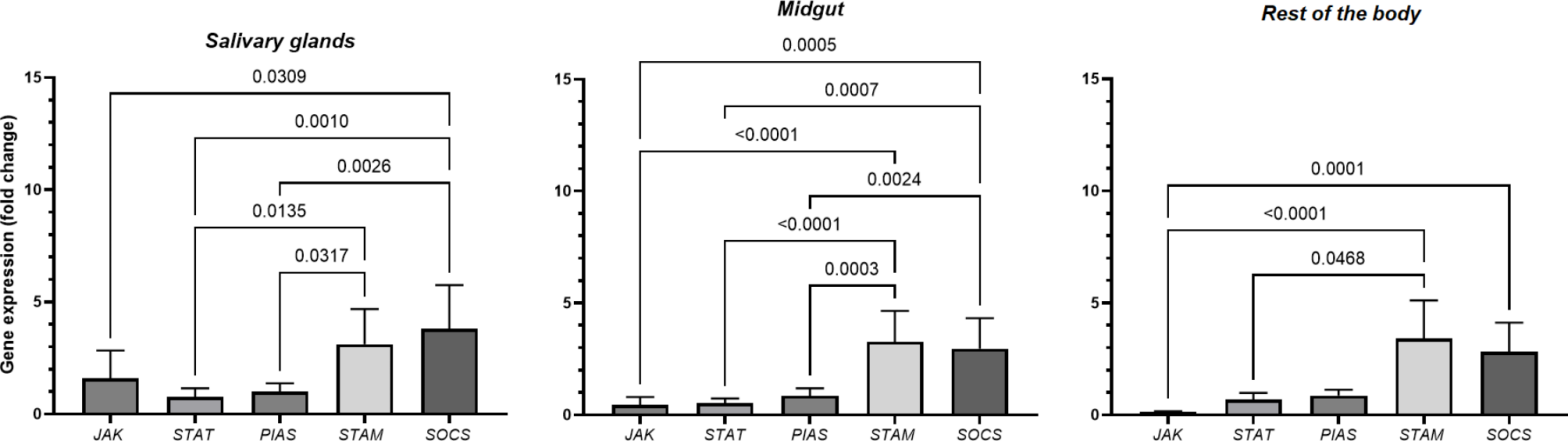
Differences in *JAK*, *STAT*, *PIAS*, *STAM*, *SOCS* expression levels in *I. ricinus* ticks with confirmed presence of *B. burgdorferi* s.l. The results shown are means and standard deviations of gene expression levels. Results in control and ticks were analysed by parametric or non-parametric ANOVA (* *p* < 0.05) and normalised to two endogenous reference genes, actin and elongation factor, and to the relative controls of the each tissue tested (relative quantification (RQ) = 1; *B. burgdorferi* s.l. negative tissue).

**JAK/STAT genes expression - Salivary glands.** Analysis of pathway gene expression in the salivary glands showed that the *STAM* gene was upregulated (3.1-fold change, *p* = 0.454). Statistical analysis revealed that the *STAM* gene was 4.1 (*p* = 0.0103), 3.1 (*p* = 0.0322) and 2.6 (*p* = 0.0020) times higher than the *STAT* and *PIAS*, respectively (Fig. 4).

**JAK/STAT genes expression - Midgut.** *SOCS* gene was observed to be expressed 9.8 (*p* = 0.0005), 5,9 (*p* = 0.0007), 3.4 (*p* = 0.0024) times higher than *JAK*, *STAT* and *PIAS* genes. The analysis showed significant differences between the *STAM* and *JAK*,* STAT* and *PIAS* genes, which were 25.4 (*p* < 0.0001), 22.5 (*p* < 0.0001) and 3.82 (*p* = 0.003), respectively (Fig. 4).

**JAK/STAT genes expression - Rest of the body.** The analysis showed significant differences between the *JAK* and *STAM*, *SOCS* genes. STAM and SOCS genes were upregulated 25.4 (*p* < 0.0001), 22.5 (*p* = 0.0001) times, respectively. The *STAM* gene was significantly more strongly expressed than the *STAT* gene (fold change: 4.5; *p* = 0.0468) (Fig. 4).

**Fig. 5:**
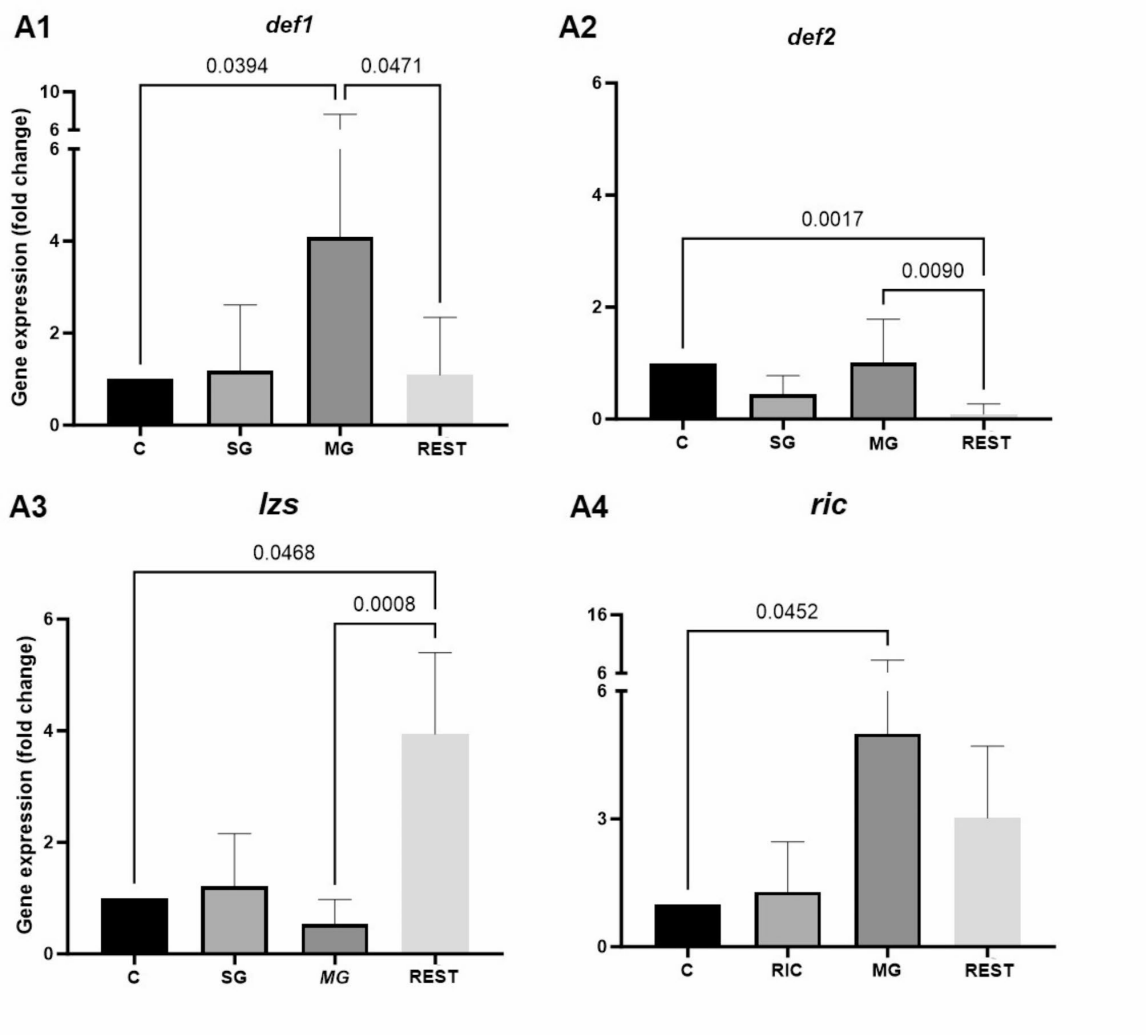
(**A**) Differences in the expression levels of *def1*, *def2*, *lzs* and *ric* in *I. ricinus* ticks with confirmed presence of *B. burgdorferi* s.l. The results shown are means and standard deviations of the gene expression levels. Results in control and ticks were analysed using parametric or non-parametric ANOVA (*p < 0.05) and normalised to two endogenous reference genes, actin and elongation factor, and relative controls (relative quantification (RQ) = 1; *B. burgdorferi* s.l. negative tissue). C- relative control of the each tissue tested, SG - salivary glands, MG - midgut, REST - the rest of the tissue after dissection.

**AMPs gene expression in ticks with confirmed presence of *B***. ***burgdorferi *s.l.** The comparison of gene expression between the tested tissues showed significant differences in all tested AMPs (Fig. 1A). The *def1* and *ric* genes were upregulated 4.1-fold (*p* = 0.0394) and 5.0-fold (*p* = 0.0452) respectively, in the midgut (Fig. 5A1, 5A4). In addition, *def1* was expressed 3.7-fold higher in the midgut than in other tissues (*p* = 0.0471) (Fig. 5A1). Expression of the *def2* gene was downregulated 11.8-fold in other tissues (*p* = 0.0017). A comparison of *def2* expression between the examined tissues showed a significant down-regulation in other tissues compared to the midgut, which was 12-fold lower (*p* = 0.0090) (Fig. 5A2). The *lzs* gene was upregulated 3.93-fold in the other tissues (*p* = 0.0468). *Lzs* gene expression was significantly higher in other tissues than in the midgut (7.45-fold, *p* = 0.0008) (Fig. 5A3).

## Discussion

Here we show how gene expression of the JAK/STAT signalling pathway and AMPs genes expression changes in the presence of *B. burgdorferi* in different tissues of questing *I. ricinus* ticks. Since transcriptomics does not always correlate with proteomics, only gene expression and not protein expression was analysed here. The research material was questing ticks. A limitation of the study is the possible influence of other microbes that might be present in the investigated individuals on JAK/STAT and AMPs expression. All individuals were collected from one geographical location and were most likely exposed to the same microhabitat. This allowed us to rule out the influence of changing environmental conditions on the expression of the genes analysed.

Activation of the JAK/STAT signalling pathway has already been observed to be triggered by bacteria, viruses or protozoa and has been shown to play a role in combating infections in ticks, whether beneficial or harmful^35^. The JAK/STAT signalling pathway is a widely expressed intracellular signal transduction pathway that plays a role in numerous biological processes, such as cell proliferation, differentiation, apoptosis, and immune regulation^36^. Rosa et al.^37^ have shown that *Anaplasma marginale* causes downregulation of immune-related genes in *Rhipicephalus microplus*. These data suggest that the pathogen can manipulate the tick’s immune system to promote its survival and colonisation in the tick vector.

**Fig. 6:**
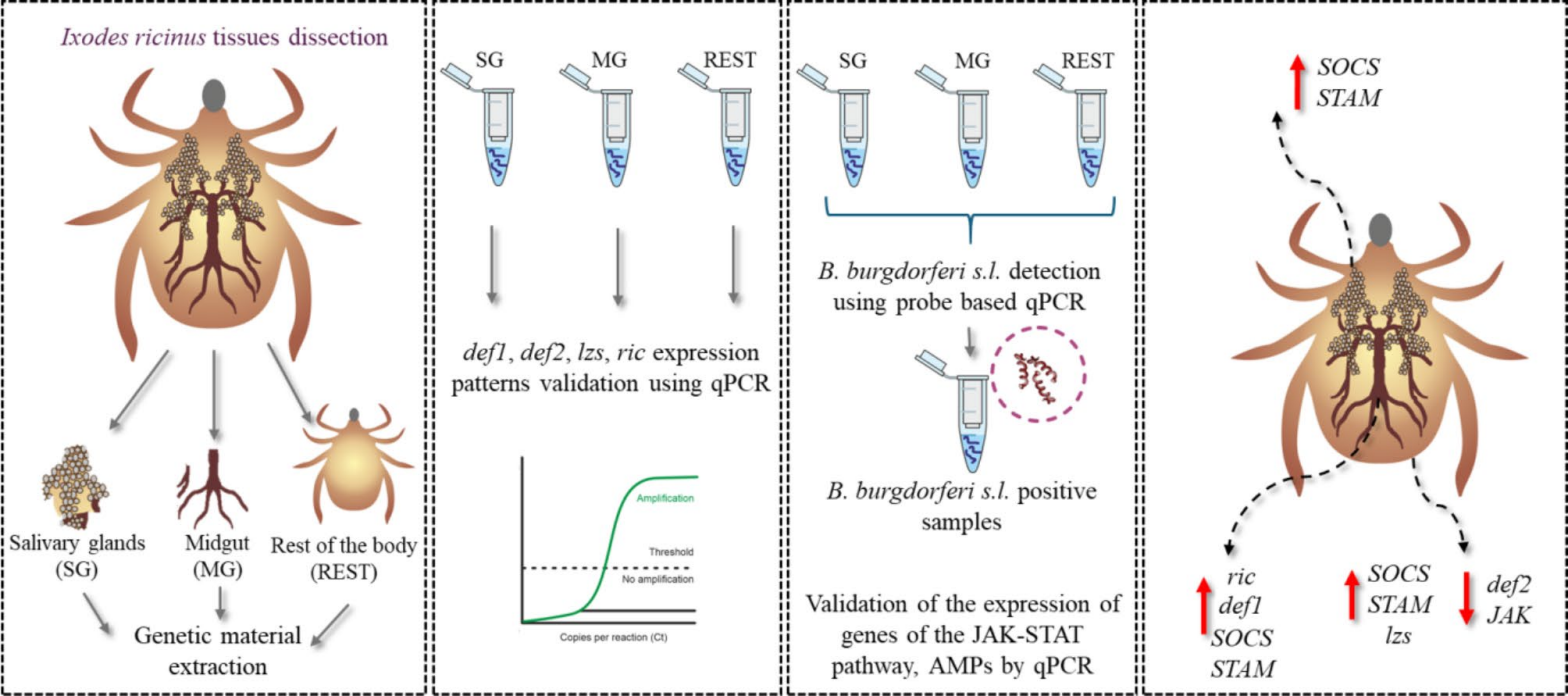
Analyses performed to determine the expression of the JAK/STAT (*JAK, STAT, STAM, SOCS, PIAS*) and AMPs (*def1*, *def2*, *lzs*, *ric*) genes expression (Made with SciPainter).

Although the tick JAK/STAT signalling pathway remains poorly understood, other reports suggest its functionality and crucial role in the control of pathogens^7–9^. Therefore, we decided to monitor the expression profile of genes related to this signalling pathway in the presence of spirochetes. We did not observe overexpression of *JAK*, *STAT* and *PIAS* genes in ticks from the natural environment that were infected with spirochetes (Fig. 6). It is known that *B. burgdorferi* s.l. spirochetes are transmitted to the host through the bite of an infected tick vector (*Ixodes*). When the tick feeds, the spirochetes located in the tick’s midgut are induced to migrate through the tick’s hemolymph to the salivary glands, from where they are then injected into the host. Although *Borrelia* bacteria may be present in the salivary glands of non-fed ticks, these bacteria do not appear to be infectious. Borreliae capable of infection regularly do not reach the salivary glands until approximately 60 to 72 h after attachment^38^. However, we found genetic material of *B. burgdorferi* s.l. in the salivary glands of the analysed questing ticks.

In this part of the study, our initial hypothesis about the overexpression of these genes during the presence of the pathogen was not confirmed. On the other hand, in all examined tissues of infected arachnids, we observed an increase in the expression of genes encoding the STAM and SOCS (Fig. 6). STAM, which is phosphorylated in response to the presence of cytokines, binds to JAKs and links cytokine stimulation to DNA synthesis^39^. We observed overexpression of the *STAM* gene in the salivary glands, midgut and rest of the tissues of *I. ricinus* (Fig. 6). We suggest the possibility of linking this to the gradual suppression of the JAK/STAT pathway during off-host period of the tick and the establishment of a balance between the immune system of the tick and that of the spirochetes. SOCS inhibit the JAK/STAT signalling pathway, resulting in reduced transcription of JAK/STAT genes^40^. Morover it can affect the continuity of signal transduction by inhibiting the catalytic activity of JAK, which initiates intracellular signalling. Thus, when SOCS is expressed at elevated levels, it may interfere with signalling transduction by inhibiting JAK^35^. In vertebrates, PIAS controls the duration of JAK/STAT signalling by causing the degradation of active STAT. In *Drosophila*, PIAS is localised in the cytoplasm and nucleus and fulfils the functions expected of a protein that regulates the degradation of activated STAT^41^. In our study, no significant changes in *PIAS* gene expression were observed. It is possible that activation of the JAK/STAT signalling pathway occurs during feeding, as in other ticks^23^.

In *I. scapularis*, studies in which the STAT and JAK transcription factors were silenced have provided evidence for the importance of the signalling pathway in controlling infection with *A. phagocytophilum*. Arthropods, like all other multicellular animals, must control the spread of invading pathogens^7^. For ticks, this is particularly challenging because they consume large quantities of blood, which can contain harmful microorganisms. To defend against infections, arthropods rely on innate immune responses that involve pathways such as Toll, Imd and JAK/STAT^10^. Cappelili-Peixoto et al.^13^ show that the survival, development and proliferation of *A. marginale* in the salivary glands and midgut cells of *R. microplu*s depend on molecular interactions between the bacterium and its biological vector. The midgut serves as the first site of interaction between ticks and pathogens and is probably the most important organ for the survival and multiplication of the pathogen^10^. On the other hand, the salivary glands are the most important site for the transmission of pathogens to the vertebrate host^13,42^. Another aim of the study was to analyse AMPs expression patterns in *I. ricinus* ticks. AMPs are key components of the innate immune system against pathogens. *Ixodes* ticks produce several AMPs. In *I. ricinus*, for example, a defensin-like gene is upregulated in the gut after infection with *B. burgdorferi*^9^. Tick defensins are generally active against Gramme-positive bacteria. Some isoforms are potentially active against Gramme-negative bacteria, protozoa, yeasts, intracellular rickettsiae, protozoa and fungi^5,43–45^. AMPs are mainly expressed in hemocytes, fat body, gut, ovaries, and salivary glands of ticks. They react to both blood-feeding and microbial stimuli, and their release into the haemolymph is crucial for an immune system response^6^. Our study confirms that in the presence of the pathogens AMPs such as *def1*, *ric*,* lzs* are overexpressed in different tissues. In this part of the study, the results obtained confirmed our research hypothesis. The highest expression level of *def1* and *def2* was found in the midgut. The *ric* gene was significantly more strongly expressed in the midgut. The expression of the *lzs* gene was almost the same in all tissues examined, only in the rest of the body it was overexpressed. We observed that the presence of borelliae induced the gene expression of defensin 1 and ricinusin in the midgut of questing *I. ricinus* females (Fig. 6). The likely increased expression of these AMPs may contribute to the silencing of infection in ticks during starvation. Similarly, Paulino et al.^35^ observed overexpression of the defensin gene in response to high load of *Theileria equi* in the intestine of *R. microplus*. Ricinusin is an antimicrobial peptide of ticks and belongs to the group of defensins, a well-preserved defence mechanism that is one of the most important components of innate immunity in ticks^46,47^. Ricinusin has been shown to be induced in *Rhipicephalus annulatus* in response to infection with *Babesia bigemina*. Although significantly higher levels of ricinusin mRNA were observed in infected ticks compared to uninfected ticks, knockdown of the gene under the conditions of this study had no effect on infection with the pathogen. This suggests that ricinusin is not critical for the control of *B. bigemina* infection in *Rhipicephalus* spp. ticks^24^. This therefore, requires further research. In rest of the body tissues, we have observed overexpression of the *lzs* gene, which encodes lysozyme. This seems to support previous studies showing that lysosomes were active in the gut of soft ticks but not in the gut of hard ticks. In the hemolymph and hemocytes of hard ticks, however the opposite is true. Here an increased expression of lysozymes was found^14^.

To summarise, AMPs are considered promising candidates for the development of novel anti-infective therapies. In arthropods such as ticks, AMPs act as the first line of defence against pathogens as part of the innate immune response^48^. On the other hand, it has been shown that the JAK/STAT signalling pathway is activated in response to pathogens and controls the expression of AMPs^6,11^. Although ticks employ a variety of molecules as antimicrobial agents, there is a notable lack of information on the regulation of their synthesis, as mentioned above. Therefore, further research on the regulation of tick AMPs by immune signalling pathways is needed to improve our understanding of their involvement in combating various pathogens^7^.

## Conclusion

The JAK/STAT pathway genes were upregulated only in the case of the *STAM* and *SOCS* genes in the salivary glands, midgut and rest of the tissues of questing *I. ricinus*. The results suggest a possible link with the gradual suppression of the JAK/STAT pathway during off-host period and the establishment of a balance between the immune system of the tick and the presence of spirochetes. AMPs have been shown to be expressed in all analysed tissues of questing ticks collected in natural environments, although there are differences between their expression patterns. There was multiple overexpression of *def1* and *ric* in the midgut. *Lzs* gene expression was significantly increased in the rest of the body tissues. We hypothesise that these AMPs may be involved in the suppression of infection during the off-host period in the midgut and the remaining tissues of the tick and that AMPs may be involved in infection management in ticks. Understanding the function and regulation of tick immune pathways is an important part of elucidating the mechanisms underlying the fitness of ticks and their vector competence. Research into their potential could lead to new strategies for managing tick-borne diseases and mitigating their impact on public health.

## Electronic supplementary material

Below is the link to the electronic supplementary material.

Supplementary Material 1

## Data Availability

The datasets used and analyzed in this study are available upon reasonable request from the corresponding author (M.S.).
