# Supplementary material for: Investigation of genes expression of the JAK/STAT signalling pathway and AMPs in the presence of Borrelia spirochetes in Ixodes ricinus
Source: Sci Rep. 2025 Jan 22;15:2869. doi: 10.1038/s41598-025-87506-6 (PMC11754740; doi:10.1038/s41598-025-87506-6)
Supplement: Supplementary file 1 — Supplementary Material 1 [file 41598_2025_87506_MOESM1_ESM.docx]

Table S1. Primers used in the study to test genes expression

| **Target Gene** | **Primer Name** | **Primer Sequence 5’-3’** | **Reference** |
| --- | --- | --- | --- |
| Actin | ACT_F | ATGTGTGACGACGAGGTTGCCGC | 15 |
|  | ACT_R | GTACAGCGACAGCACGGCCTGG |  |
| Elongation factor | EF_F | ACGAGGCTCTGACGGAAG | 27 |
|  | EF_R | CACGACGCAACTCCTTCAC |  |
| *I.ricinus*_def1 | Def1_F | GGTGGCTACTACTGCCCATTTTTT | 15 |
|  | Def_R | TCAGACGCAGATGCAGGTCTTTT |  |
| *I. ricinu*s_def2 | Def2_F | GGTGGTTACTACTGCCCATTCCG | 15 |
| *Lysozyme* | LSZ_F | CTGGTTTCCGCCCATACACT | present study |
|  | LSZ_R | CTCTCTCCACGATCGCAGAC |  |
| *Ricinusin* | RIC_F | CGCTGAGAACGAAACTGCAA | present study |
|  | RIC_R | ACTGACAGTGCTCAACGGTT |  |
| *JAK* | JAK_F | AACCGCAAGCTGCCTATGAA | present study |
|  | JAK_R | CGAGGTTCTCCTGAGTCACG |  |
| *STAT* | STAT_F | AGGTCAAGGTGTCCATCATC | 10 |
|  | STAT_R | GATACTCCATTGTTCCTGTGTTG |  |
| *STAM* | STAM_F | GCTCAGGAGACATCTCAGCC | present study |
|  | STAM_R | GATACAGGCTCTTGGCAGGG |  |
| *PIAS* | PIAS_F | GAGGCGAGTGATCCTGCAAT | present study |
|  | PIAS_R | GCCCTCACATACCCACTCAC |  |
| *SOCS* | SOCS_F | CTGTTCCACCCAGCAGAGAG | present study |
|  | SOCS_R | CGGCTCTACGATAACCGGAC |  |
